# Early Palliative Care in Advanced Hematologic Malignancies: A Systematic Review of Patient-Centered Outcomes

**DOI:** 10.3390/healthcare13212789

**Published:** 2025-11-03

**Authors:** Patrícia Fernandes-Almeida, Paulo Reis-Pina

**Affiliations:** 1 Center for Palliative Medicine, Faculty of Medicine, University of Lisbon, 1649-028 Lisbon, Portugal; 2 Acute Palliative Care Inpatient Unit, Local Health Unit of Santa Maria, 1649-028 Lisbon, Portugal

**Keywords:** attitudes to death, blood transfusion, facilities and services utilization, health care costs, hematologic neoplasms, hospice care, palliative care, quality of life, symptom burden

## Abstract

**Highlights:**

**What are the main findings?**

- Early palliative care in hematologic malignancies improves symptoms and quality of life, while reducing hospitalizations, transfusions, and chemotherapy near the end-of-life.
- Early referral is associated with lower healthcare costs and a shift in the place of death from hospital to home or hospice.

**What is the implication of the main finding?**

- These results support integrating early palliative care into standard hematology practice.
- Wider implementation could improve patient outcomes, reduce burdensome treatments, and optimize healthcare resource use.

**Abstract:**

**Background:** Patients with hematologic malignancy (HM) experience high symptom burden (SB) and diminished quality of life (QOL). While early palliative care (EPC) benefits solid tumors, its impact in HM remains uncertain. **Objectives:** To systematically review the effects of EPC on patient-centered outcomes in individuals with HM. **Methods:** MEDLINE, Web of Science, Cochrane Library, and Scopus were searched for English-language articles published between 2020 and 2024. Eligible studies included adults with advanced HM receiving EPC compared with usual care, reporting outcomes on SB, QOL, place of death (POD), healthcare costs (HCCs), or healthcare utilization (HCU). All original study designs were considered. Critical appraisal was performed, and results were synthesized narratively. The review was registered in PROSPERO (CRD420251019687). **Results:** Twelve studies were included, most of high quality (n = 10) and mainly conducted in America and Europe. Collectively, they enrolled 42,053 participants, largely with advanced disease, poor performance status, or limited prognosis. EPC consistently improved SB, particularly pain, appetite, and functional well-being, although results for anxiety and depression were inconsistent. Findings for QOL were mixed. EPC was associated with higher likelihood of home or hospice death. One study demonstrated substantial cost savings with home-based EPC. Across several studies, EPC was linked to lower HCU, including fewer transfusions, reduced chemotherapy near the end-of-life, and fewer aggressive interventions, hospitalizations, and intensive care admissions. **Conclusions:** EPC improves SB, influences POD, and reduces HCCs and HCU in HM. Evidence for QOL and psychological outcomes remains inconclusive. Further high-quality research is required to consolidate these findings.

## 1. Introduction

### 1.1. Rationale

Cancer is a major global health concern, with incidence rates rising worldwide. Hematologic malignancy (HM) accounts for around 6.5% of all cancers globally, and about 9.0% in both the United States of America and Europe [1]. The National Institutes of Health defines HM as cancers that originate in blood-forming tissue, such as the bone marrow, or in the cells of the immune system. This heterogeneous group of diseases displays substantial variation in illness trajectories, treatment strategies and potential for cure [2].

Historically, HM has been at the forefront of cancer treatment innovation, resulting in improved therapeutic outcomes and survival rates. Nevertheless, many patients continue to experience significant symptom burden, adverse effects of chemotherapy, relapsed disease, and ultimately death from their malignancy. These challenges make them appropriate candidates for early palliative care (EPC), either alongside or independent of active cancer treatment, to achieve effective symptom control and enhance quality of life (QOL) [3,4].

The concept of palliative care (PC) has evolved in recent years. Once focused primarily on end-stage cancer, PC now encompasses the care of all life-limiting conditions [5]. The World Health Organization defines PC as an approach that improves the QOL of patients and their families facing life-threatening illness, through early identification, comprehensive assessment, and management of pain and other physical, psychological, and spiritual problems [6]. This multidisciplinary model promotes well-being at any stage of disease, regardless of prognosis [6].

PC has been associated with improved QOL, reduced symptom burden, and decreased psychological distress [3]. However, patients with HM are less likely to access PC than those with solid tumors and are more likely to receive aggressive EOL care [7,8,9,10], and to die in intensive care units (ICU) [7]. Patients with HM are disproportionately likely to die in hospital, yet emerging evidence suggests that EPC may reduce hospital deaths and increase home/hospice deaths—shown in other serious illnesses—supporting its relevance to place of death (POD) in HM [4,11,12]. Emerging evidence also suggests that EPC can lower healthcare costs (HCCs)—particularly with early inpatient or home-based models and near the end-of-life (EOL)—although effects may vary by timeframe and setting [13,14,15,16,17]. Additionally, EPC is associated with reduced healthcare utilization (HCU)—e.g., shorter time per patient, fewer transfusions, less chemotherapy, and fewer ICU admissions, hospitalizations, and emergency department (ED) visits [13,18]—with similar patterns reported in other serious illnesses [11,15,19,20].

Several barriers to PC integration in HM have been identified, including the difficulty in defining the terminal phase of these diseases [21], common misconceptions equating PC with EOL care [22], strong patient-physician relationships within hematology teams, and the continuous emergence of novel treatments [23]. As a result, PC research in HM lags that of solid tumors. Additional investigation is needed to clarify the role of EPC in this patient population.

### 1.2. Objectives

This study aimed to systematically review and synthesize the available evidence on the associations between EPC and patient-centered outcomes among individuals with HM. Specifically, it examined how EPC relates to QOL, symptom control/burden (SCB), POD, HCU, and HCCs in this population.

## 2. Methods

This systematic review was conducted in accordance with the “Cochrane Handbook for Systematic Reviews of Interventions” [24], which informed the development of the search strategy, study selection, data extraction, critical appraisal, and synthesis processes. Reporting followed the “Preferred Reporting Items for Systematic Reviews and Meta-Analyses” (PRISMA) guidelines [25].

A protocol was developed before screening and guided all stages of the review. The protocol was registered with PROSPERO (CRD420251019687); registration was submitted on 25 March 2025 and accepted on 26 March 2025, after title/abstract screening had begun but before full-text data extraction and synthesis. No changes were made to the prespecified objectives, eligibility criteria, outcomes (QOL, SCB, POD, HCU, HCCs), or synthesis plan after registration.

### 2.1. Eligibility Criteria

Studies were selected based on the following criteria:

*Participants*: Adults (≥18 years) with advanced HM (e.g., leukemia, lymphoma, myeloma).

*Intervention*: EPC delivered concurrently with or in addition to active cancer treatment. Any definition of “early palliative care” as provided by the original study authors was accepted.

*Comparator*: Usual care without early integration of PC, including delayed or no PC.

*Outcomes*: Primary outcomes: QOL and SCB; Secondary outcomes: POD, HCCs, and HCU.

*Study Design*: All study designs were considered, except for reviews, editorials, letters, opinion pieces, academic theses, protocols without results, congress posters or abstracts reporting only preliminary findings, and commentaries.

Only English-language records were eligible. Full texts unavailable via our institutional subscriptions were excluded. We did not pursue interlibrary loans or contact authors for access.

### 2.2. Information Sources

We searched MEDLINE, Web of Science, Cochrane Library, and Scopus for articles published between 11 September 2020, and 19 July 2024. All databases were last searched on 20 July 2024. The search start date was chosen because a prior systematic review included studies through 10 September 2020 [26]. Building on that work, the present review focuses specifically on EPC timing (as opposed to specialty PC in general) and updates the evidence through July 2024 with pre-specified primary outcomes (QOL, SCB) and secondary outcomes (POD, HCU, HCCs). This scope allows us to examine whether earlier PC integration is associated with differences in patient-centered outcomes and resource use beyond what was previously reported. An updated review within a short interval was justified by (i) the emergence of home-based/embedded EPC models and digital electronic patient-reported outcomes (e-PRO) platforms in HM care after 2020; and (ii) publication of newer studies (e.g., large utilization analyses, clinic-based EPC cohorts) with endpoints aligned to our pre-specified outcomes (QOL, SCB, POD, HCU, HCCs). Collectively, these developments warranted a focused EPC update beyond the timeframe and scope of Elliott et al. [26].

### 2.3. Search Strategy

The search strategy included the following terms: (early OR timely OR refer*) AND (“palliative care” OR “hospice care” OR “end of life care” OR “terminal care”) AND (“hematolog* malignanc*” OR “blood cancer*” OR “bone marrow cancer” OR leukemia OR lymphoma OR myeloma) NOT review. Filters were applied for: Language: English; Population: Adults aged ≥ 19 years; Publication date: 11 September 2020, to 19 July 2024.

The full electronic search strategy for all four databases is presented in Supplementary Table S1.

### 2.4. Selection Process

**Figure 1:**
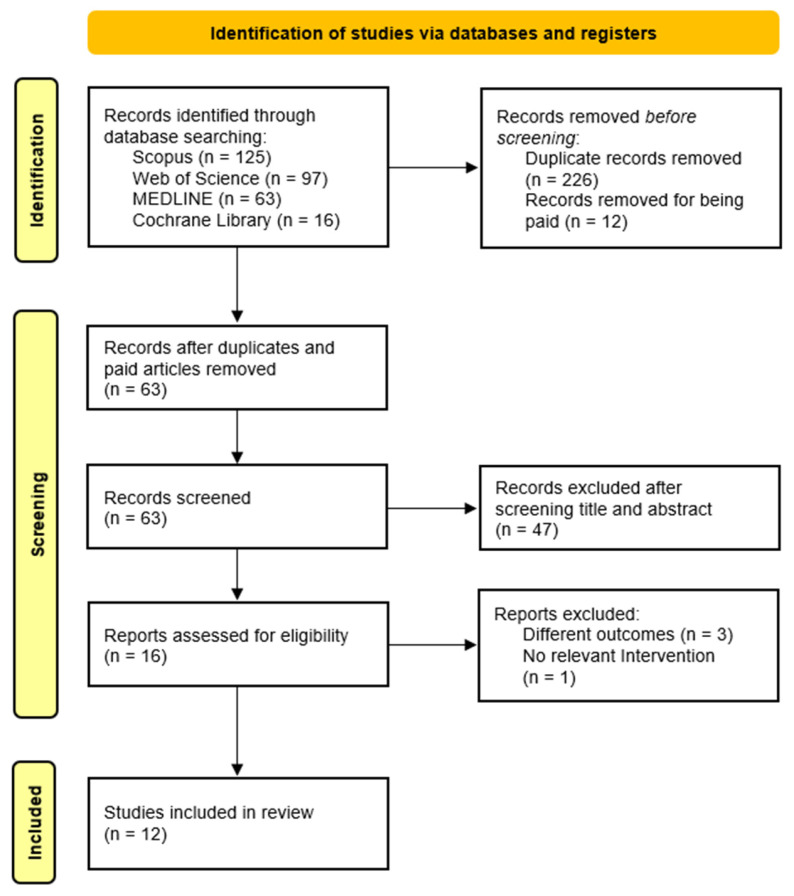
Flow diagram of the study selection process.

Following the initial search using the predefined headings and filters, two independent researchers screened the titles and abstracts of identified articles. Full texts were then reviewed for eligibility based on study design, objectives, population, and outcomes. Studies meeting all inclusion criteria were selected for review. Any disagreements during study selection or data extraction were resolved through discussion and consensus between the authors. See Figure 1 for the study selection process.

### 2.5. Data Collection Process

Two reviewers independently extracted data using a standardized, pre-piloted extraction form (spreadsheet template). The form captured: bibliographic details; country/setting; study design; population/disease subtype; sample size and follow-up; EPC model and timing; comparator; outcomes (QOL, SCB, POD, HCU, HCCs); measurement tools/scales; and key results relevant to the review question. After independent extraction, records from each study were compared side-by-side. Any differences in content or coding were discussed and resolved by consensus, with reference to the full text and prespecified definitions. A simple change log was maintained to document reconciliations and corrections. For verification, one reviewer cross-checked all critical numeric fields (e.g., sample sizes, counts, effect numbers reported in text/tables/figures) against the source article; the second reviewer verified corrections before finalization. No automation tools were used, and authors were not contacted for additional information. No formal inter-rater reliability statistic (e.g., kappa) was calculated; full agreement was reached through discussion.

### 2.6. Data Items

Data were collected for the following outcomes: QOL, SCB, POD, HCCs, and HCU. All outcome-related results reported in each study, irrespective of the measurement tools used, were included. Additional variables extracted comprised article characteristics (authors, country of origin, year of publication); study design and objectives; intervention details and duration; participant characteristics and type of HM; outcomes assessed, scales/tools applied, and key findings. In addition, we recorded how “early” or “timely” PC was defined by the original authors.

### 2.7. Study Risk of Bias Assessment

Two reviewers independently assessed the risk of bias for each included study. After independent evaluation, the reviewers compared their assessments and discussed any discrepancies in judgment or scoring. Differences were resolved through structured discussion and mutual agreement, referring back to the study report and the appraisal tool criteria when needed. No third reviewer was involved, and no formal inter-rater statistic was calculated. For randomized controlled trial(s) (RCT), the Cochrane Risk of Bias 2 (RoB 2) tool was used [27]. For non-randomized studies, the ROBINS-I tool was applied [28]. The Critical Appraisal Skills Programme (CASP) checklists were used to appraise cohort studies [29], cross-sectional [30], and qualitative studies [31]. Case reports were assessed using the JBI Critical Appraisal Checklist [32]. Each domain was evaluated according to the guidance and algorithms of the respective tools. No automation tools were used.

### 2.8. Effect Measures

For each outcome, the effect measures reported by the original study authors were accepted and used in both the synthesis and presentation of results.

### 2.9. Synthesis Methods

Given the limited number of eligible studies and the substantial heterogeneity in participants, interventions, and outcomes, neither meta-analysis nor meta-regression was undertaken. Instead, the evidence was synthesized narratively. No formal evidence grading framework was applied.

Definitions of EPC were extracted and interpreted as reported by the original authors, with attention to timing relative to diagnosis and patients’ clinical context. To enhance clarity, data were charted, grouped, and synthesized across the following categories: definitions of EPC, QOL, SCB, POD, HCCs, and HCU. Additionally, a summary across outcomes was prepared to concisely integrate the main patterns emerging across all outcomes, highlighting consistent and divergent findings.

**Figure 2:**
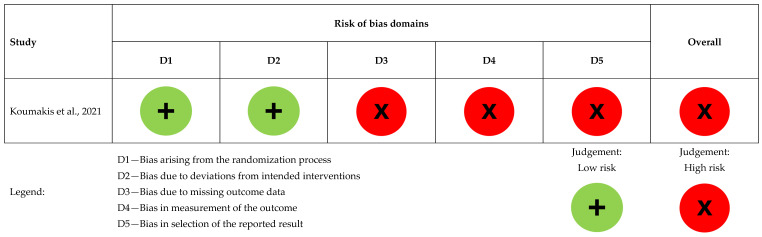
Risk of bias assessment for the randomized controlled study (n = 1), [5].

**Figure 3:**
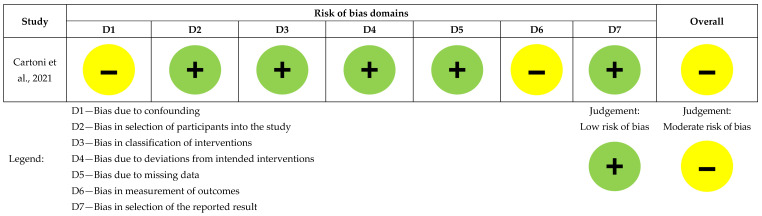
Risk of bias assessment for the non-randomized comparative study (n = 1), [13].

We used four standardized, pre-piloted charting/extraction forms (Excel templates) to ensure consistent recording and reporting:

1. Study Characteristics form—bibliographic details; country/setting; design; population/HM subtype; sample size and follow-up; EPC model and timing; comparator. (Feeds Table 1)
2. EPC Operationalization form—working definition of EPC in each study; EPC components (e.g., symptom management, goals-of-care discussions); team composition; care setting (inpatient/outpatient/home); frequency/intensity; timing criteria. (Feeds Table 2)
3. Outcome Extraction form—for each prespecified outcome (QOL, SCB, POD, HCU, HCCs): instrument/scale used, measurement timepoints, direction of effect, and numerical results where available (estimates/percentages/events). (Feeds Table 3)
4. Risk of Bias form—design-specific item checklists mirroring the criteria of the appraisal tool used for each design, plus an overall judgement and rationale. (Feeds Figure 2 and Figure 3, and Table 4)

All forms were piloted on a subset of studies and refined prior to full extraction. Two reviewers completed the forms independently and reconciled discrepancies by discussion to consensus (see Section 2.5).

## 3. Results

### 3.1. Study Selection

The initial search across the four databases yielded 301 records. After removing duplicates (n = 226) and excluding inaccessible articles (n = 12), 63 articles remained. Following title and abstract screening, 47 articles were excluded. Sixteen full-text articles were then assessed for eligibility, of which four were excluded: three for not reporting relevant outcomes and one for lacking an appropriate intervention. In total, 12 studies met the inclusion criteria and were included in this systematic review. The study selection process is illustrated in Figure 1.

### 3.2. Study Characteristics

The characteristics of the included studies are summarized in Table 1.

In this review, most studies originated from America, specifically from the United States [33,34,35,36,37] and Brazil [3]. One study was multicentric, including participants from both the United States of America and Italy [18]. In Europe, studies were conducted in Denmark [38] and Italy [39]. Two additional multicentric European studies included Austria and Italy [13] and Czechia, Germany, Greece, Italy, and the United Kingdom [5]. Finally, one study was from Asia, namely China [23].

We included six observational studies—four retrospective [3,18,23,34], one prospective [36], and one cross-sectional [35]—as well as two case studies [38,39], one RCT [5], one mixed-methods case study [37], one non-randomized comparative study [13], and one qualitative study [33].

Across the 12 included studies, the overall sample comprised 42,053 participants. This number is strongly influenced by the largest study [34], which reported 41,789 hospitalizations of patients with diffuse large B-cell lymphoma. Excluding this outlier, the remaining 11 studies included 264 patients, caregivers, or cases.

Populations mainly involved adults with acute myeloid leukemia (n = 5), multiple myeloma (n = 5), myelodysplastic syndromes (n = 5), and non-Hodgkin’s lymphomas (n = 3). Across studies, patients often had advanced disease stages, poor performance status, or limited prognosis (n = 7).

### 3.3. Risk of Bias in Studies

Koumakis et al. had a high overall risk of bias. Randomization was low risk, but due to ongoing MyPal trials, only validation results were available, limiting the assessment of other domains [5], as presented in Figure 2.

Cartoni et al. had low risk across all domains except for confounding and outcome measurement bias [13], as seen in Figure 3.

Risk of bias for nine studies assessed using CASP checklists is summarized in Table 4, with two studies [37,39] assessed for both qualitative and cohort studies.

Chan et al. and Henckel et al. were assessed with low risk across all domains [23,33].

For Weisse et al. the overall quality was moderate due to selection/referral bias, small cohort size, and limited generalizability, with a predominance of women and high median age, potentially limiting applicability to broader populations [37].

Weisse et al. and Bigi et al. were evaluated using qualitative criteria, with all domains marked ‘yes,’ except for the researcher-participant relationship, marked ‘can’t tell‘ due to insufficient detail [37,39].

In the quantitative assessments of Ebert et al. and Bigi et al., confounding factors were not controlled [3,39].

Richter et al. had low risk of bias, except for cohort recruitment, due to selection bias from excluding severely ill individuals [35].

Samala et al. had incomplete follow-up (60%), small cohort size, and demographic limitations, reducing generalizability, resulting in mixed bias ratings [36].

Jackson et al. had follow-up issues, as rehospitalizations could not be tracked due to data limitations [34].

Potenza et al. acknowledged retrospective limitations and single-center design, marking some domains as ‘can’t tell’ for confounding and applicability [18].

The case report by Sørensen et al. [38] had low risk of bias, with all domains marked ‘yes’ (see Table 4).

### 3.4. Results of Individual Studies

The main results of individual studies for the outcomes included in this review are presented in Table 3.

### 3.5. Results of Syntheses

#### 3.5.1. Early Palliative Care in Patients with Hematologic Malignancies

Based on our interpretation of the definitions of EPC only five studies were judged to have implemented truly EPC, as shown in Table 2. In three studies, the information provided was insufficient to determine whether the intervention qualified as early. In the remaining studies, there was no clear intervention to classify, or PC was delivered in a hospice context, making it incompatible with typical definitions of EPC.

#### 3.5.2. Quality of Life

Five studies addressed QOL [5,33,35,36,38]; however, only two–both of high methodological quality–specifically evaluated the impact of EPC on QOL [36,38]. In Sørensen et al.’s case study involving a patient with a newly diagnosed Multiple Myeloma (MM), no significant improvement in QOL scores was observed over a 10-week period following the first specialized PC consultation [38]. Conversely, Samala et al. reported a notable improvement in overall QOL after 12 months of EPC involvement in patients with newly diagnosed MM [36].

#### 3.5.3. Symptom Control/Burden

Two studies focused on symptom burden [13,39]. Four studies addressed pain [18,23,36,38], two investigated depression and anxiety [23,36], one assessed appetite [23], and one evaluated multiple domains of well-being [36].

##### Symptom Burden

Regarding symptom burden, Cartoni et al., in a moderate risk of bias study comparing early home PC with standard hospital care, reported no significant differences between groups [13]. However, these findings should be interpreted cautiously, as patients in the early home PC group were generally older, frailer, and had a poorer prognosis.

Bigi et al., in a community case study of HM patients referred to two EPC units, did not provide a direct comparison between early and late referral groups at the Carpi Unit but observed that patients tended to report improvements in symptom burden regardless of referral timing [39].

##### Symptom Control

In four studies assessing pain, EPC was associated with improvements in pain scores over time [18,23,36,38]; contributing studies showed low or some concerns for risk of bias.

Chan et al., in a retrospective study of 38 patients with advanced HM, observed a significant improvement in mean symptom scores for depression and anxiety after the fourth follow-up [23]. Conversely, Samala et al. reported no significant changes in depression or anxiety scores over time [36].

Chan et al. also demonstrated a significant improvement in appetite scores after the fourth follow-up [23].

Samala et al. reported improved functional well-being but found no significant changes in physical, social, or emotional well-being subscales [36].

#### 3.5.4. Place of Death

This outcome was addressed in two studies [18,39]; contributing studies had low risk of bias or some concerns.

Bigi et al. evaluated an EPC program across two units. In the Carpi unit, patients were more likely to die at home regardless of the timing of referral to PC. In the Modena unit, similar findings were reported, with 50.7% of patients dying at home or in a hospice and only 5.3% dying in an acute care facility. However, the study did not analyze the relationship between timing of referral and POD in the Modena unit [39].

Potenza et al. reported that patients receiving EPC were more likely to die at home or in hospice rather than in hospital or acute care settings [18].

#### 3.5.5. Healthcare Costs

Only one study addressed this outcome. Bigi et al. concluded that early home PC was less expensive than standard hospital care, resulting in a weekly saving of 2314.90 euros for the healthcare provider. Additionally, it was found to be cost-effective, with an incremental cost-effectiveness ratio of 7013.90 euros per prevented day of care due to avoided infections [39].

#### 3.5.6. Healthcare Utilization

This outcome was assessed in three studies [13,18,39].

One study showed that the mean weekly time per patient dedicated by physicians and nurses was lower in the EPC group compared to standard hospital care [13].

Two studies reported a reduced use of transfusions among patients receiving EPC [13,18].

Two studies found that late PC referrals were associated with greater use of chemotherapy [18,39].

Potenza et al. also reported higher opioid use in patients receiving early supportive/PC. Additionally, these authors found that patients with early supportive/PC had lower HCU, including fewer interventions such as intubation and cardiopulmonary resuscitation, as well as reduced ED visits, ICU admissions, and multiple hospitalizations [18].

Taken together, the findings across studies reveal common patterns despite heterogeneity in design, population, and outcome measures. While individual results are detailed above, the following summary synthesizes the overall direction and consistency of effects across the prespecified outcomes—QOL, SCB, POD, HCU, and HCCs—providing a concise overview of the main signals emerging from the evidence base.

### 3.6. Summary Across Outcomes

- SCB: EPC improved symptom control in three studies, particularly pain and related domains (numerical improvements over time in pain; gains in energy/appetite reported) [18,23,36].
- QOL: Findings were mixed. One study showed significant QOL improvement at 12 months [36]; another suggested potential QOL gains with a digital e-PRO platform [5]; a case report showed stable QOL [38].
- HCU: EPC was associated with reduced utilization in ≥3 studies—fewer transfusions, less chemotherapy near EOL, and fewer ICU admissions/ED visits/multiple hospitalizations [13,18,39].
- HCCs: Lower costs were reported in one comparative study of early home PC vs. hospital care [13].
- POD: Two studies showed a shift toward home/hospice deaths with earlier referral/integration [18,39].
- Survival: Signals were inconsistent/limited; one program reported higher 1-year survival with EPC compared with delayed referral [39], while other data were descriptive or not EPC-specific.

Axial/theme: EPC was consistently linked with fewer aggressive EOL markers (e.g., less late chemotherapy, ICU, ED/multiple admissions) and greater alignment with preferred care settings (home/hospice), with pain improvement the clearest patient-level benefit; QOL results were heterogeneous across instruments and designs.

Across studies, several outcomes tended to co-occur. Improvements in SCB—particularly pain relief and functional well-being—were frequently accompanied by reduced HCU and a shift in POD toward home or hospice settings [13,18,39]. These patterns suggest that earlier EPC integration may simultaneously influence both patient-centered outcomes and indicators of healthcare intensity, although causal inferences cannot be drawn.

## 4. Discussion

### 4.1. Summary of Findings

Across 12 studies, EPC improved SCB (n = 6)—notably pain, appetite, and functional well-being—while results for anxiety and depression were mixed. For QOL (n = 5), findings were mixed. EPC reduced HCU (n = 3)—including fewer transfusions, less late chemotherapy, and fewer aggressive interventions—and lowered HCC in one study (home-based EPC). EPC was associated with a shift in POD toward home or hospice (n = 2). These patterns support timely referral to EPC in HM.

### 4.2. Early Palliative Care in Patients with Hematologic Malignancies

Only five studies met the criteria for genuine EPC, defined as the integration of PC early in the disease trajectory. There is no consensus on the timing that qualifies a PC intervention as “early,” with definitions varying across studies. In available interventional research, EPC is often defined as an intervention delivered within 8 to 12 weeks of diagnosis. For example, Vergnenègre et al. considered EPC to be initiated within three months of diagnosis in their prospective observational study [40], consistent with Hui et al. who adopted a similar timeframe in their retrospective cohort analysis [9]. In contrast, other studies aligned with the 2016 American Society of Clinical Oncology guidelines, which defined EPC as an intervention occurring within 8 weeks of diagnosis [41,42].

Recent updates to the American Society of Clinical Oncology guidelines have shifted away from strict time-based definitions [43]. Instead, they recommend referring patients with advanced cancer to specialized, interdisciplinary PC teams early in the course of the disease, alongside active cancer treatment. The guidelines emphasize that “early” should not be interpreted as waiting until cessation of antineoplastic therapy, but rather as responding to the presence of palliative needs [43].

EPC has also been defined by other parameters, including the setting of the intervention, such as outpatient versus inpatient consultation [44], or by the duration of continuity of care before death (e.g., >90 days, 31–90 days, 11–30 days, and 1–10 days) [45].

In a recent scoping review that investigated how EPC was defined in the literature for adults with life-limiting illnesses, definitions for EPC were organized in five categories: time-based, prognosis-based, location-based, treatment-based and symptom-based [46]. Among patients with cancer, most EPC definitions described were time-based and the majority of studies considered EPC when the patients were diagnosed with advanced cancer within the previous 6–8 weeks. Differently, in multiple or non-cancer diagnosis the most common definition category was symptom-based [46].

These variations underscore the absence of a universally accepted definition of EPC and the complexity of operationalizing it in research and clinical practice.

While a growing body of literature demonstrates the many benefits of EPC in adults with advanced cancer, evidence supporting its role in HM remains limited and warrants further investigation. There are barriers to equitable access to PC, including difficulties in identifying patients nearing the EOL, the “survival imperative,” the “normalization of dying,” misconceptions, mistrust, limited information about PC and EOL care, and a fragmented care system [47].

Although the integration of PC into cancer care represents a patient- and family-centered, interdisciplinary approach recommended throughout the disease trajectory, its early implementation remains limited; delays in referral–often reducing PC to EOL interventions–may reflect a persistent lack of knowledge, training, and preparedness among healthcare professionals to address serious illness and the dying process, raising ethical concerns about the preservation of human dignity [48].

Several studies have identified potential causes for delayed PC referral in this population [2,10,49,50,51,52,53,54]. One study categorized these barriers into three broad domains: cultural, illness-specific, and system-based [2]. Cultural barriers include misconceptions equating PC solely with EOL care and a general lack of awareness about its broader benefits throughout the disease trajectory. Illness-specific barriers stem from the unpredictable and complex nature of HM, the aggressive and prolonged treatments involved, and prognostic uncertainty, all of which complicate the timing of PC integration. System-based barriers often include limited availability of EOL care services and constraints in delivering blood products in hospice settings [2].

Late referrals, when patients are already significantly ill, limit the time available for PC teams to establish a therapeutic relationship and implement effective interventions. These delays may reduce the overall impact and benefits of PC in this population. To address these barriers and enhance the integration of PC, several strategies have been proposed.

One effective approach involves rebranding PC as “supportive care”, a change shown to reduce stigma and substantially increase early referrals. In one study, early PC referrals rose from 43% to 81% among solid tumor specialists and from 21% to 66% among hematologic specialists following this terminology shift [55]. Salins et al. similarly emphasized the importance of renaming PC as “supportive care” as a key recommendation from oncologists [56]. Other enablers of equitable and timely referral to PC include thorough patient evaluation, addressing basic survival needs and social determinants of access, and fostering intersectoral collaboration, community advocacy, and engagement [47].

Beyond terminology, improving awareness and fostering positive attitudes toward PC are essential to promote timely referrals [52]. This may be achieved by incorporating PC training into hematology and oncology residency programs and ensuring that PC providers possess cancer-specific knowledge and skills [56]. These steps are crucial to strengthening the integration of PC into standard cancer practice.

### 4.3. Early Palliative Care and Quality of Life

In our systematic review, Samala et al. reported that in patients with newly diagnosed MM, EPC involvement over 12 months led to an improvement in overall QOL [36]. In contrast, the case report by Sørensen et al. did not show any changes in QOL scores following EPC intervention [38]. The inconsistency in QOL findings may be explained by the use of different assessment instruments across studies, each measuring distinct domains and applying non-equivalent scoring systems.

The literature on the impact of EPC on QOL in patients with HM remains scarce. However, several studies in other patient populations have demonstrated benefits. Temel et al., in an RCT involving patients with metastatic Non-Small Cell Lung Cancer (NSCLC), showed that early PC integration significantly improved QOL, reduced depressive symptoms, led to less aggressive EOL care, and even prolonged survival [57]. Similar findings were observed in another RCT by Chen et al. also involving NSCLC patients, where EPC was associated with extended survival, improved QOL, greater psychological stability, reduced pain, and better nutritional satisfaction [58]. Additional studies have also supported the positive impact of EPC in patients with advanced cancer across a range of outcomes [59,60,61,62,63]. Nonetheless, the evidence is not unanimous, either for cancer or non-cancer patients. For instance, Allende et al., in an RCT conducted in Mexico, found no significant differences in QOL or symptom burden between EPC and standard oncological care in patients with metastatic NSCLC [64]. Similarly, a recent review reported no significant QOL differences between EPC and usual care in patients with heart failure, end-stage liver disease, chronic obstructive pulmonary disease, or idiopathic pulmonary fibrosis [20].

### 4.4. Early Palliative Care and Symptom Control/Burden

The literature consistently shows that patients with HM experience a high symptom burden [26,65], stemming not only from the disease itself but also from the aggressive treatments often administered near the EOL [7,8,9]. Consequently, these patients are clear candidates for PC, which can improve QOL through a multidisciplinary approach that addresses physical, spiritual, and psychosocial needs. Given these benefits, PC should be considered a standard component of care for this population. However, several studies indicate that patients with HM are less likely to receive PC or hospice care compared to patients with solid tumors [4,8,53]. Moreover, when PC referrals do occur, they are frequently delayed, often taking place in the final days of life [26,66].

This issue was evident in one of the studies included in our systematic review, conducted by Ebert et al., which reported that patients with HM, particularly those with MM and acute leukemia, presented with significant symptom burden at the time of their first PC consultation [3]. Notably, the study revealed limited and delayed access to PC services, especially for patients with acute leukemia; only 18 of 97 patients with this diagnosis were referred to PC during the study period [3].

Due to such delays, the evidence on the impact of EPC on symptom burden in HM remains limited. In our review, six studies assessed SCB, with improvements reported in pain [18,23,36,38], depression and/or anxiety [23], and appetite [23]. However, in the prospective cohort study by Samala et al., depression and anxiety scores did not significantly change over time [36].

A recent study comparing EPC to usual hematologic care in patients newly diagnosed with MM supports the integration of EPC into standard care. It demonstrated that pain intensity significantly decreased over time in the EPC group, but not in the control group [67].

Haun et al., in a systematic review, concluded that EPC may improve QOL and symptom intensity in patients with advanced cancer compared to standard care. Although effect sizes were small, they may still be clinically meaningful in late-stage disease. However, levels of depressive symptoms did not differ significantly between patients receiving EPC and those receiving usual care [68].

Additional studies involving patients with advanced cancer have also demonstrated the positive effects of EPC on symptom burden [59,61,62]. Nonetheless, some studies [59,61,62] found no significant differences, specifically in anxiety [59,61] or depression [61].

To conclude this part of the discussion, it is important to recall the evidence concerning patients with advanced non-cancer diseases who also often experience late referral to PC due to prognostic uncertainty. A 2025 systematic review on EPC in non-oncological populations found that EPC reduced anxiety and depression in stroke patients and improved pain interference and fatigue in those with heart failure. However, findings on anxiety and depression in heart failure patients were inconsistent [20].

### 4.5. Early Palliative Care and Place of Death

A systematic review and meta-analysis by Howell et al. revealed that patients with HM are more than twice as likely to die in hospital compared to those with other types of cancer, despite home being widely recognized as the preferred setting for EOL care [4].

While limited evidence directly links EPC to POD in HM populations, findings from other serious illnesses suggest a positive impact. For example, a single-center retrospective observational study by Rodrigo-Troyano et al., involving patients with interstitial lung diseases, demonstrated that EPC referral was independently associated with a reduced risk of hospital admissions during the last year of life and a lower likelihood of dying in hospital [11]. Similarly, Bassi et al. reported that initiating an EPC program at the time of diagnosis significantly reduced hospital death rates, instead increasing the likelihood of dying at home or in hospice settings [12].

### 4.6. Early Palliative Care and Healthcare Costs

HCCs were addressed in one study included in this review, which suggested that early home PC for patients with HM was less expensive than standard hospital care [13]. Supporting this, a prospective cohort study conducted at a large academic medical center found that patients who received early inpatient PC consultations had a significantly greater reduction in total HCC compared to those who received later referrals [14]. Specifically, EPC patients saw an average, and statistically significant, decline of $1431 in total costs in the 1-day pre/post consultation period, compared to a $403 reduction in the late PC cohort. Over a 3-day period, the total cost reduction was $5839 for the EPC group versus $1478 for the late PC group (*p* < 0.001) [14].

In other serious illnesses, similar trends were observed. For instance, in end-stage liver disease, a retrospective review found that hospitalization costs were significantly lower among those referred early to PC referrals [15].

Conversely, a retrospective study involving both cancer and non-cancer patients found that earlier involvement of specialist PC was associated with higher overall HCC during the last year of life. However, in the final 1 and 3 months of life costs were lower in the PC group, largely due to reduced hospitalizations [16]. This trend was also confirmed by Seow et al., who reported decreased healthcare expenditures in the last month of life among cancer patients receiving PC, again primarily attributed to fewer hospital admissions [17].

### 4.7. Early Palliative Care and Healthcare Utilization

This outcome was assessed in three studies. Overall, the findings indicated that EPC was associated with reduced HCU. This was reflected in shorter time spent per patient [13], lower rates of blood transfusions [13,18], chemotherapy [13,18], intubation, cardiopulmonary resuscitation, fewer ICU admissions, multiple hospitalizations, and fewer ED visits [18]. Only one study reported a higher use of opioids among patients receiving early supportive/PC [18], which is not surprising given that opioids are essential for effective symptom management in PC settings.

The broader literature on HCU shows similar trends. For example, in patients with end-stage liver disease, EPC referral was associated with significantly fewer endoscopies and blood transfusions [15]. In interstitial lung diseases, EPC was independently associated with a lower risk of hospital admissions in the last year of life [11]. Bevins et al. also reported fewer ED visits and reduced ICU admissions in patients with pancreatic cancer who received EPC [19]. A recent review showed that EPC positively influenced time to first readmission and increased days alive outside the hospital among patients with end-stage liver disease [20].

However, not all studies confirm these findings. In an RCT involving patients with advanced cancer, Bakitas et al. found no significant differences between early and delayed PC groups in terms of HCU. This included hospital days, ICU days, ED visits, chemotherapy in the last 14 days of life, and rates of home death [69]. Similarly, Vanbutsele et al., in another RCTs did not observe significant differences in HCU between the intervention and control groups [63].

### 4.8. Strengths and Limitations

This systematic review has several strengths. It is one of the few reviews that specifically examines the impact of EPC in patients with HM, addressing an important but underexplored area in palliative hematology/oncology. A rigorous search strategy was applied across multiple databases, and the review was conducted in accordance with PRISMA guidelines, including a critical appraisal of the identified studies.

The review evaluates a wide range of outcomes, including QOL, SCB, HCU, HCCs, and POD, thereby providing a comprehensive overview of EPC’s potential benefits in this population. It includes findings from diverse study designs—such as RCTs, cohort studies, and case studies—conducted across different healthcare systems, which enhances the breadth and depth of interpretation. Beyond clinical outcomes, it also critically explores cultural, disease-specific, and system-level barriers to EPC integration, offering valuable practical insights for implementation. Furthermore, this review contributes to raising awareness of an underexplored area in HM, a field traditionally characterized by late PC referrals and aggressive EOL treatments. By synthesizing the benefits and challenges of EPC, it underscores the urgent need for further research and for integrating EPC into standard hematologic care.

Nevertheless, several limitations must be acknowledged. This review received no funding, which restricted access to some paid articles and may have reduced the comprehensiveness of the evidence base. The number of studies addressing EPC in HM remains limited, and much of the higher-quality evidence derives from populations with solid tumors, reducing generalizability to HM. The included studies were heterogeneous in design, sample size, disease subtypes, timing and structure of EPC interventions, and outcome measures, precluding meta-analysis and limiting comparability. Many were observational, introducing risks of bias such as selection bias and non-standardized treatment protocols, while the lack of RCT restricts the ability to draw firm causal inferences.

Additional methodological constraints should also be considered. Language and access restrictions may have introduced selection and publication bias. We limited inclusion to English-language publications and excluded studies whose full texts were inaccessible via our institutional library; we did not request interlibrary loans or contact authors. These constraints may bias the evidence toward settings and journals with greater English-language indexing and subscription availability and may underrepresent studies from low- and middle-income countries or non-English contexts. Accordingly, estimates of effect and generalizability should be interpreted with caution. Moreover, if the search was limited to specific databases, important studies may have been inadvertently omitted. PROSPERO registration occurred after screening had started—although before data extraction and synthesis—which is suboptimal for transparency; however, no post hoc changes were made to objectives, eligibility, outcomes, or the synthesis plan. Citation tracking and reference list screening were not performed, and no formal evidence grading framework was applied, which reduces both the interpretability and the clinical applicability of the findings. Finally, there is still no consensus on standardized definitions or models of what constitutes “early” PC, complicating the assessment of its true impact.

### 4.9. Implications for Practice, Policy, and Future Research

Practice. In adults with HM, EPC appears to improve SCB—particularly pain and functional well-being—based on multiple contributing studies (see Results: SCB). Findings for QOL were mixed (Results: QOL). EPC was associated with a shift in POD toward home/hospice in limited studies (Results: POD) and with lower HCU (e.g., fewer transfusions, less late chemotherapy, fewer ICU/ED/hospital admissions) in several studies (Results: HCU), with cost-saving signals in one comparative analysis (Results: HCCs). Clinicians should consider timely referral to EPC when symptom load escalates or at diagnosis of advanced disease, coordinated closely with hematology to align goals of care and symptom management (Results: Summary across outcomes).

Policy. Health systems should facilitate timely access to EPC in hematology by resourcing integrated/embedded models and by tracking patient-centered outcomes (SCB, QOL) alongside care-intensity metrics (POD, HCU, HCCs) reflected in our review (Results: POD/HCU/HCCs). Given mixed QOL findings and heterogeneous tools, policies should also promote standardized outcome measurement across services (Results: QOL).

Future Research. Priorities include the following: (i) prospective comparative designs/RCT where feasible to strengthen causal inference; (ii) evaluations of optimal timing and models (embedded, home-based, e-PRO-enabled); (iii) robust economic analyses (HCCs) and linkage with HCU/POD; and (iv) harmonized, validated QOL/SCB instruments to reduce measurement heterogeneity that may obscure effects (Results: QOL). Multicenter studies in diverse settings are needed to improve generalizability.

## 5. Conclusions

Building on prior evidence for specialty PC in HM [26], this EPC-focused update (2020–2024) suggests that earlier integration appears to improve SCB—particularly pain—and may reduce aggressive EOL indicators, HCU, and HCCs, with a shift in POD toward home/hospice in limited studies. Findings for QOL and psychological outcomes were mixed, likely influenced by heterogeneous instruments. Late PC referral remains common and likely attenuates potential benefits. Future rigorously designed studies should clarify causal effects and identify which EPC components (including embedded/home-based and ePRO-enabled models) yield the greatest benefit in HM.

## Figures and Tables

**Table 1:** Characteristics of the included studies (n = 12).

Authors, Year, Country	Study Design	Objectives	Interventions	Duration	Participants	Hematologic Diseases	Outcomes	Scales or Tools	Key Observations
Ebert et al., 2023, Brazil [3]	Retrospective cohort study.	Characterize symptom burden, performance status and clinical characteristics of a cohort of HM patients referred to PC outpatient consultation.	PC consultation.	3 years and 9 months.	59 HM patients (30 men, median age (years old): 71 (AML), 54 (NHL high grade), 75 (NHL low grade) and 73 (MM).	AML, NHL (high and low grade) and MM.	Symptom burden, major symptoms, median time from diagnosis to first PC appointment, median time from the first PC consultation until death, median overall survival from diagnosis.	PPS, ECOG Performance Status, ESAS.	-Only 3 patients diagnosed with Hodgkin’s lymphoma were referred to the PC service. -Only 1 patient with primary myelofibrosis and 1 patient with MDS during study time range were referred to the PC service.
Koumakis et al, 2021, Czechia, Germany, Greece, Italy and UK [5]	Research project, two-arms RCT.	Evaluate MyPal’s feasibility and its potential impact on QOL of PC patients with HM.	MyPal platform: specialized applications supporting the regular answering of standardized questionnaires, symptoms reporting, educational material provision and notifications.	1 year: first 6 months providing e-PRO information + 6 months follow-up with monthly questionnaires.	-Adult patients with CLL or SLL or MDS, scheduled to receive any line of treatment or who have been previously exposed to any treatment -Life expectancy > 3 months.	CLL, SLL or MDS.	QOL	EORTC QLQ-C30 General Questionnaire, EuroQOL EQ-5D, IPOS, ESAS, BPI and Emotion Thermometer.	-MyPal is a Horizon 2020 European project aiming to support PC for cancer patients via the e-PRO, (patient’s self-assessed health status) to gauge efficacy of treatment. -Patients’ recruitment was still ongoing, so results are from the validation phase; however, MyPal project was completed on 31 December 2022 and an eBook (with study design and preliminary outcomes) was published.
Cartoni et al., 2021, Austria and Italy [13]	Non-randomized comparative study.	Compare costs, resources, and clinical outcomes between an early home PC program and standard hospital care for active-advanced or terminal phase patients.	Early home PC for patients with HM that include symptom control, psychosocial care, serious illness conversations, and an individualized management plan appropriate for optimizing patients’ QOL and reducing caregivers’ burden.	1 year and 2 months.	-119 adults (62 men, mean age of 66.1 years), diagnosed with a HM. -KPS ≤ 60.	AML, ALL, MDS or other HM.	Rate of new infections and hemorrhages, mean weekly number of erythrocyte or platelets transfusions, symptom burden, mean weekly cost of care, cost-minimization difference and ICER.	KPS, MDASI	-This program included a multidisciplinary team (physicians, nurses, psychologists, and social workers) trained in hematology, PC, and supportive care. -Allocation was based on the caregiver availability; travel time to hospital < 60 min; and psychosocial situation and home environment suitable for home care program.
Potenza et al., 2024, Italy and USA [18]	Observational, retrospective study.	Investigate quality indicators for PC and EOL care in patients with AML receiving early supportive PC.	-Early supportive PC (symptom control, support for decision making, future planning, coping facilitation, physical and emotional support). -The PC team also assesses patients’ prognostic awareness.	5 years and 8 months.	215 AML patients (118 men) consecutively enrolled at a hematology early supportive PC clinic in Modena, Italy.	AML	EOL care, treatments near EOL (ChT, ICU admission, intubation, opiate use, red cell or platelet transfusions), QOL, timing of PC.	N/A	N/A
Chan et al., 2021, China [23]	Retrospective study.	Evaluate the clinical outcomes from the early integrated PC model for patients with advanced HM.	Early integrated PC.	2 years and 4 months.	38 advanced HM patients (22 men, mean age 70.5 years).	AML, MDS or other HM.	Symptom burden, pharmacological interventions used by PC.	ESAS	The significant symptom is defined as the ESAS score ≥ 4.
Henckel et al., 2020, USA [33]	Qualitative study.	Characterize the perspectives of HM patients and bereaved caregivers on the utility of hospice services and transfusion access with respect to QOL.	A semi-structured focus group guide with open-ended questions to elicit perspectives regarding QOL, existing or desired supportive care services, and transfusion access.	8 months.	27 adults: 18 patients (12 males) with estimated life expectancy ≤ 6 months); and 9 bereaved caregivers (8 females) whose loved ones passed away between 3 and 12 months prior.	Leukemia, MDS, lymphoma and myeloma.	Association of traditional hospice services and transfusion with QOL, physical and functional well-being, pain, desire for energy and fatigue.	N/A	N/A
Jackson et al., 2023, USA [34]	Retrospective cohort study.	Examine the prevalence of PC utilization and evaluate the predictors of PC receipt among patients with DLBCL.	N/A	3 years.	41789 hospitalizations of adult patients diagnosed with DLBCL; and 2973 uses of PC (55.60% were male)	DLBCL	Prevalence of PC utilization, predictors of PC utilization (age, gender, ethnicity, insurance type, hospital location, patient disposition, admission type, length of stay, ChT and number of comorbidities).	CCI	N/A
Richter et al., 2021, USA [35]	Cross-sectional study.	Determine the prevalence of symptom burden and psychological distress, and the association of distress with survival.	N/A	1 year and 2 months.	239 patients (137 men, median age 67 years) with MM.	MM	Performance status, pain, financial and family burden, depression, distress, overall survival.	CPASS-7	The CPASS-7 is a statistically validated tool that evaluates distress from the point of view of the patient with advanced cancer.
Samala et al., 2024, USA [36]	Prospective cohort design.	Determine the effects and the feasibility of EPC integration on MM patients.	EPC integration on patients with newly diagnosed MM.	1 year	-20 adult patients (5 men, median age 65 years), within 8 weeks of diagnosis of active symptomatic MM. -ECOG 0–3.	MM	QOL, FWB, pain, depression and anxiety.	FACT-MM, HADS, Physical, social, emotional and FWB subscales and pain subscale.	FACT-MM is a 41-item measure of health-related QOL for patients with MM receiving anti-neoplastic treatment.
Weisse et al., 2024, USA [37]	Retrospective convergent mixed-methods case study.	Understand the EOL care experiences of hospice patients with HM when death occurs in a residential care setting.	Hospice care.	15 years (registry of hospice patients who died at RCH between 2005 and 2020).	-35 patients: 18 with HM (7 men and median age of 81.5 years), and 17 with solid tumors (7 men and median age of 83 years) -Prognosis of ≤3 months -Unable to access home hospice due to housing or caregiver instability.	Leukemia, lymphoma, MM	EOL symptom management, skin integrity, bleeding, bone pain, delirium, HM-directed palliative medications at the EOL, psychosocial support and overall quality of death.	N/A	-Out of 535 hospice patients who died, only 29 with HM, but just 18 had narratives available and medication review. -Social hospice model RCH: managed by community members who provide custodial care for patients unable to receive hospice care at home, due to housing or caregiver instability.
Sørensen et al., 2022, Denmark [38]	Case study.	Description and discussion of the course of a patient with newly diagnosed MM.	Early specialized PC.	13 weeks.	49-year-old man with newly diagnosed MM, involving all vertebrae and with no common analgesic treatment providing sufficient relief.	MM	Pain relief, anxiety, total distress.	EORTC QLQ C15-Pall symptom screening tool, HADS.	EORTC QLQ C15 is an abbreviated 15-item version of the EORTC QLQ C30, a cancer health-related QOL questionnaire.
Bigi et al., 2023, Italy [39]	Community case study.	Document the results of a long term clinical and research experience in delivering EPC service to address both solid and blood cancer patients’ and their primary caregivers’ needs.	-EPC program (symptom assessment and management, support in decision making, future planning, facilitation of coping, physical and emotional support, and patients’ prognostic awareness). -Periodic tutorial meetings with oncologists, hematologists and nurses.	4 years.	292 advanced cancer patients (most of them with solid tumors) in Carpi; and 215 patients with HM in Modena.	AML, MM and other high-risk HM.	Morbidity, mortality, symptom burden; ChT, blood transfusions and referral to ICU near the EOL.	ESAS	The provision of EPC took place in two EPC units, one in Carpi and the other in Modena.

Legend: AML—acute myeloid leukemia; ALL—acute lymphoblastic leukemia; BPI—brief pain inventory; CCI—Charlson comorbidity index; ChT—chemotherapy; CLL—Chronic lymphocytic leukemia; CPASS-7—Cota Patient Assessed Symptom Score-7 item; DLBCL—Diffuse Large B-Cell Lymphoma; ECOG—Eastern Cooperative Oncology Group; EOL—End of life; EORTC QLQ Cn—European Organization for Research and Treatment of Cancer Core Quality of Life questionnaire (*n* = number of items); EPC—early palliative care; e-PRO—electronic patient-reported outcomes; ESAS—Edmonton Symptom Assessment Scale; EuroQOL EQ-5D—Health-related QOL (5 Dimensions); FACT-MM—Functional Assessment of Cancer Therapy–Multiple Myeloma; FWB—Functional well-being; HADS—Hospital Anxiety and Depression Scale; HM—hematologic malignancy; ICER—incremental, cost-effectiveness ratio; ICU—intensive care unit; IPOS—Integrated Palliative care Outcome Scale; KPS—Karnofsky Performance Scale; MDASI—M.D. Anderson Symptom Inventory; MDS—Myelodysplastic syndrome; MM—multiple myeloma; N/A—Not available; NHL—non-Hodgkin’s lymphoma; PC—palliative care; PPS—Palliative Performance Scale; QOL—Quality of life; RCH—Residential care home; SLL—small lymphocytic lymphoma; USA—United States of America.

**Table 2:** Definition of “early palliative care” in the included studies (n = 12).

Authors, Year, Country	What Do Authors Say?	Our Interpretation
Ebert et al., 2023, Brazil [3]	Although no explicit definition of what constitutes an EPC intervention is provided, the results report the median time (in months) from diagnosis to the first PC appointment: -AML: 5 (range 1–21); -high grade NHL: 7 (range 0–17); -low grade NHL: 8 (range 2–22); -MM: 9 (range 0–44).	Given that patients across all four clinical entities were referred to their first PC appointment in a timely manner-within 0 to 2 months after diagnosis, it is reasonable to conclude that the PC intervention was implemented as “early” as feasible within the given clinical context.
Koumakis et al, 2021, Czechia, Germany, Greece, Italy and UK [5]	This study aimed to enhance the QOL of patients with HM by introducing PC early in the disease trajectory. Authors refer that the trial is still ongoing, so the validation results are the only results available.	There is insufficient information to confirm that this qualifies as a true EPC intervention.
Cartoni et al., 2021, Austria and Italy [13]	Authors mention a specific program of domiciliary provision of supportive and EPC for patients, that for descriptive purposes were categorized as being in an active-advanced or terminal phase. Critical aspects of this program include symptom control, psychosocial care, serious illness conversations, and an individualized management plan appropriate for optimizing patients’ QOL and reducing caregivers’ burden.	There is insufficient information to confirm that this qualifies as a true EPC intervention.
Potenza et al., 2024, Italy and USA [18]	The ePSC intervention was started on the same day as the very first hematological outpatient visit and the intervention was defined early when provided within 8 weeks from cancer diagnosis. The results reported a median time from AML diagnosis to first ePSC outpatient visit of 5 weeks (range 0–21 weeks). Furthermore, authors considered a full ePSC intervention when patients with AML received ≥3 visits in the ePSC clinic and patients with only one or two visits were considered late referrals.	Given the median time from AML diagnosis to first ePSC outpatient visit at 5 weeks (range 0–21 weeks), it is reasonable to conclude that the PC intervention was implemented as “early” as feasible within the given clinical context.
Chan et al., 2021, China [23]	Patients with advanced HM could be referred to the PC team after failing two or more lines of treatment, as they are considered truly refractory patients. Referral could also be made earlier, following failure of first-line therapy, if poor prognostic indicators are present, such as frail elderly, poor functional status or significant complications due to disease treatment.	It is reasonable to conclude that the intervention was not delivered within the context of hospice care or EOL care, but there is insufficient information to confirm that this qualifies as a true EPC intervention.
Henckel et al., 2020, USA [33]	Not defined.	The intervention was delivered within the context of hospice care.
Jackson et al., 2023, USA [34]	Not defined.	The objective of the study was to examine the prevalence of PC utilization and evaluate the predictors of PC receipt among patients with DLBCL, so there is no intervention to classify as early or not.
Richter et al., 2021, USA [35]	Authors only mention other studies, the majority of which have been in solid tumors, where PRO assessments were used to assess the clinical benefit of EPC interventions.	There is no intervention to classify as early or not.
Samala et al., 2024, USA [36]	Patients were enrolled within eight weeks of diagnosis. Participants met with the PC team within three weeks of enrollment. Visits usually concluded with the establishment of a PC plan comprised of medication initiation and/or adjustment, referral to other healthcare providers and advance care planning. Participants met with the PC team at least once a month for twelve months.	Although there is no specific reference to the definition of EPC, it is reasonable to conclude that the PC intervention was implemented as “early” as feasible within the clinical context, occurring approximately 3 months after diagnosis.
Weisse et al., 2024, USA [37]	This study described the EOL experiences of patients with HM who died while receiving routine hospice home care.	The intervention was delivered within the context of hospice care.
Sørensen et al., 2022, Denmark [38]	Specialized PC is a multidisciplinary need-based approach from the time a life-threatening disease is diagnosed. The authors describe the case of a 49-year-old man, previously healthy, that was admitted to the department of hematology after symptoms and findings of MM, confirmed with a bone marrow biopsy. After this, a specialized PC interconsultation was requested on day 4 of admission.	It is reasonable to conclude that the intervention qualifies as a true EPC intervention.
Bigi et al., 2023, Italy [39]	Authors state that patients admitted at the EPC unit in Carpi were assigned to either EPC or “delayed palliative/supportive care” groups, based on the time elapsed between the diagnosis and the initiation of the PC, using 90 and 60 days as a cut-off in a primary and secondary analysis, respectively. At the Modena Unit, PC was defined early when patients received ≥ 3 visits and delayed when <3 visits. However, they also mention that in both cases, the PC intervention was defined as “early” when provided within 8 weeks from cancer diagnosis.	The authors appear to use different criteria to EPC, including the number of visits patients received and the time from diagnosis. Based on the criterion of timing we can conclude that the intervention qualifies as a true EPC intervention.

Legend: AML—acute myeloid leukemia; DLBCl—diffuse large B cell lymphoma; EOL—end of life; EPC—early palliative care; ePSC—early palliative supportive care: HM—hematologic malignancy; MM—multiple myeloma; NHL—non Hodgkin’s lymphoma; PC—palliative care; PRO—patient-reported outcomes; QOL—quality of life; USA—United States of America.

**Table 3:** Main findings of the individual studies (n = 12).

Authors; Country; Year	Group Qualities	Quality of Life	Symptom Burden	Timing and Use of Palliative Care	Healthcare Utilization and Costs	Survival	Place of Death
Ebert et al., 2023, Brazil [3]	AML	-Median PPS score of 70 -Median ECOG was 2	High ESAS scores for pain (5), tiredness (7), depression (5) and well-being (5).	-Median time from: diagnosis to first PC appointment—5 months; the first PC consultation until death—3 months. -Median of 2 PC appointments before death.	N/A	Median overall survival from diagnosis: 14 months	N/A
	High grade NHL	-Median PPS score of 70 -Median ECOG was 2	High ESAS score for anxiety (5).	-Median time from: diagnosis to first PC appointment—7 months; the first PC consultation until death—4 months. -Median of 3 PC appointments before death.	N/A	Median overall survival from diagnosis: 11 months	N/A
	Low grade NHL	-Median PPS score of 50 -Median ECOG was 2	High ESAS score for anxiety (5).	Median time from: diagnosis to first PC appointment—8 months; the first PC consultation until death—10.2 and 5.1 months (only 2 patients had died until data analyses).	N/A	Median overall survival from diagnosis: 16.8 and 18.8 months (only 2 patients had died until data analyses).	N/A
	MM	-Median PPS score of 50 -Median ECOG was 3	High ESAS score for pain (7), tiredness (6) and anxiety (6).	-Median time from: diagnosis to first PC appointment—9 months; the first PC consultation until death—4 months. -Median of 3 PC appointments before death.	N/A	Median overall survival from diagnosis: 18 months	N/A
Koumakis et al, 2021, Czechia, Germany, Greece, Italy and UK [5]	N/A	A digital platform for PC, such as MyPal, can improve the patient’s QOL and communication between patients and physicians.	N/A	N/A	N/A	N/A	N/A
Cartoni et al., 2021, Austria and Italy [13]	Early home PC	Patients were more debilitated than the patients in the hospital group.	-Symptom burden was similar in both groups. -Rate of infections: 21.20%. -New hemorrhages: 8.50%.	N/A	-Mean weekly number of transfusions: 1.45. -Mean weekly time per patient dedicated by physician and nurse: 270 and 235 min, respectively. -Costs related to health professionals were the highest (~60%) and were significantly higher compared with hospital care (~20%). -Average MWC for provider: 1219.7 euros -Average MWC for patient/family: 162.6 euros. ICER cost odds for avoided days of care: −7013.9 euros	Median overall survival: 2.75 months.	N/A
	Standard hospital care	Patients had a higher ADL score, higher Hb levels and a lower MDASI symptom interference score at baseline.	-Symptom burden was similar in both groups. -Rate of infections: 53.70%. -New hemorrhages: 11.70%.	N/A	-Mean weekly number of transfusions: 2.77. -Mean weekly time per patient dedicated by physician and nurse: 340 and 600 min, respectively. -Costs for drugs were the highest (~45%). -Average MWC for provider: 3534.3 euros. -Average MWC for patient/family: 76.7 euros.	Median overall survival: 8.39 months.	N/A
Potenza et al., 2024, Italy and USA [18]	Early supportive PC	N/A	There was a statistically significant improvement in pain intensity over time (median NRS improving from 4, at baseline, to 0 after 12 weeks).	-The median time from AML diagnosis to first EPC outpatient visit was 5 weeks (range 0–21 weeks). -Only 13 out of 131 were first referred to EPC clinic more than 8 weeks from diagnosis.	-2.7% of patients received ChT in the last 14 days of life. -None underwent intubation or CPR or was admitted to ICU during the last month of life. -Only 4% had either multiple hospitalizations or two or more ED access. -58.7% received opioids within 30 days of death. -49.3% and 41.3% of patients did not receive a red cell or platelet transfusion, respectively, within 7 days of death.	N/A	-Hospice or home: 50.7%. -Hospital: 44%. -Acute facility: 5.3%
	Late PC	N/A	N/A	N/A	-13.9% received ChT within 14 days of death. -6.1% underwent intubation and 14.7% of patients were admitted to ICU in the last month of life. -11.8% had > 2 hospitalizations and 23.5% had > 2 access to ED within 30 days of death. -40.5% received opioids within 30 days of death. -28.12% and 28.16% of patients did not receive a red cell or platelet transfusion within 7 days of death, respectively,	N/A	-Hospice or home: 30.6%. -Hospital: 69.4%. -Acute facility: 31.4%
Chan et al., 2021, China [23]	N/A	N/A	At baseline: -50–60% reported significant symptoms (ESAS score ≥ 4): fatigue, anxiety, drowsy, and anorexia. -42% had significantly depressed moods. -37% had pain. After 4th follow-up, the mean symptoms scores improved for pain (*p* = 0.017), depression (*p* = 0.023), anxiety (*p* = 0.003), and appetite (*p* = 0.007).	N/A	Most prescribed medications were used in 30–50% of patients, and included appetite stimulants, opioids, and antidepressants.	N/A	N/A
Henckel et al., 2020, USA [33]	N/A	-Both transfusions and traditional hospice were important for QOL. -The absence of pain was not vocalized in any of the patient groups when defining QOL. -Only one caregiver noted that pain management was important for the QOL.	-Patients with blood cancers reported greater levels of fatigue compared with other patients. -Improvements in energy, dyspnea, and the ability to engage in activities they enjoyed were reported as benefits of transfusions.	N/A	N/A	Patients and caregivers expressed that transfusions were necessary for survival.	N/A
Jackson et al., 2023, USA [34]	N/A	N/A	N/A	-7.1% used PC during hospitalization, while 4.8% utilized PC and were discharged alive. -DLBCL patients aged > 70 years had 1.3 times higher odds of using PC. -Patients who were transferred to a facility, discharged with home health or died during hospitalization had higher odds of receiving PC, compared with patients with routine hospital discharge. -Patients electively admitted to the hospital had 30% lower odds of receiving PC. -Patients who received ChT had 62% lower odds of having a PC consultation. -Patients with CCI > 8 were 1.4 times more likely to use PC, compared to those with CCI of 0 to 4.	N/A	N/A	N/A
Richter et al., 2021, USA [35]	N/A	-48% were concerned that they could not do things they wanted. -33% reported decreased performance status. -Financial burdens were 44%, with 5% experiencing severe financial toxicity. -24% with Family burden.	-15% with depression. -41% lacked pleasure. -36% with concerns. -24% with high total distress score and trended toward an association with a decreased survival rate compared to the 182 patients (76%) with a low total distress score.	N/A	N/A	The 6-month survival rates for patients with high and low distress scores were 86% and 96%, respectively, and the 12-month survival rates were 76% and 87%, respectively.	N/A
Samala et al., 2024, USA [36]	N/A	Overall QOL improved after 12 months of EPC involvement.	-Measures significantly improved at 12 months: FACT-G scores by 15.1 points, FWB scores by 6.0 points and Pain Subscale scores by 3.4 points. -Subscale scores for PWB, SWB, EWB, total FACT-MM and HADS scores for both depression and anxiety did not significantly change overtime.	N/A	N/A	N/A	N/A
Weisse et al., 2024, USA [37]	N/A	N/A	-Patients with HM exhibited common EOL symptoms (pain, dyspnea and agitation). -50% with skin integrity issues and 4 patients had skin lesions and/or edema. -For 78% no bleeding occurred; however, narratives reflected the high risk of bleeding. -22% admitted with fractures, with increased pain. -Most with some degree of cognitive impairment and/or delirium. -78% with good symptom management and peaceful or comfortable deaths.	N/A	-Medications aimed at managing pain, dyspnea, terminal restlessness or delirium, secretions, constipation and nausea/vomiting. -There were no significant differences in liquid morphine utilization and LOS between the HM and ST cohorts.	N/A	Patients died at RCH and were peaceful or comfortable and accompanied by family and friends.
Sørensen et al., 2022, Denmark [38]	N/A	Both in the first specialized PC interconsultation and 10 weeks after discharge, the patient reported a QOL score of 4 (1 = very poor, 7 = very good).	-In the first specialized PC interconsultation, the patient reported severe pain, reduced level of function, insomnia, weakness, constipation, fatigue and tension. -10 weeks after discharge, the patient reports pain as being ‘‘quite a bit’’ and all other symptoms were reported as ‘‘mild’’ or ‘‘none at all’’.	N/A	N/A	N/A	N/A
Bigi et al., 2023, Italy [39]	EPC unit in Carpi- EPC group	N/A	Regardless of timing of PC referral, patients were more likely to report improved symptom burden and mood.	N/A	Frequency of ChT use in the last 60 days of life: 3.4%	Estimated survival probability at 1 year: 74.5%	Regardless of timing of PC referral, patients were more likely to have home deaths.
	EPC unit in Carpi- delayed PC group	N/A		N/A	Frequency of ChT use in the last 60 days of life: 24.6%	Estimated survival probability at 1 year: 45.5%%	
	EPC unit in Modena	N/A	N/A	N/A	-Frequency of ChT use in the last 14 days of life: 2.7%. -No patients were admitted to the ICU during the last month of life. -49.3% and 41.3% received red cell or platelet transfusions, respectively, within 7 days of death.	N/A	-50.7% died at home or in a hospice. -5.3% died in an acute facility.

Legend: ADL—Activities of Daily Living; AML—acute myeloid leukemia; CCI -Charlson comorbidity index; ChT—chemotherapy; CPR—Cardiopulmonary resuscitation; DLBCL—Diffuse Large B-Cell Lymphoma; ED—emergency department; EOL—end of life; EPC—early palliative care; ePSC—early palliative supportive care; ESAS—Edmonton Symptom Assessment Scale; EWB—Emotional Well-Being; FACT-G—Functional Assessment of Cancer Therapy–General; HADS—Hospital Anxiety and Depression Scale; Hb—hemoglobin; HM—hematologic malignancy; ICER—incremental cost-effectiveness ratio; ICU—intensive care unit; LOS—length of stay; MDASI—MD Anderson Symptom Inventory; MM—multiple myeloma; MWC—mean weekly cost of care; N/A—Not available; NHL—non-Hodgkin’s lymphoma; NRS—Numerical rating scale; PC—palliative care; PPS—Palliative Performance Scale; PWS—Physical Well-Being; QOL—quality of life; SPC—specialized palliative care; ST—solid tumors; SWB—Social Well-Being; USA—United States of America.

**Table 4:** Risk of bias assessment of observational, case report, and qualitative studies (n = 10).

Authors, Year of Publication, Country	Tool	Total Number of Items	Number of “Yes”	Number of “No”	Number of “Can’t Tell”	Degree of Quality	Overall Appraisal
Ebert et al., 2023, Brazil [3]	CASP	14	13	0	1	High	Include
Potenza et al., 2024, Italy and USA [18]	CASP	14	11	0	3	High	Include
Chan et al., 2021, China [23]	CASP	14	14	0	0	High	Include
Henckel et al., 2020, USA [33]	CASP	10	10	0	0	High	Include
Jackson et al., 2023, USA [34]	CASP	14	12	0	2	High	Include
Richter et al., 2021, USA [35]	CASP	11	10	0	1	High	Include
Samala et al., 2024, USA [36]	CASP	14	12	1	1	High	Include
Weisse et al., 2024, USA–outcome 1 [37]	CASP	10	9	0	1	High	Include
Weisse et al., 2024, USA–outcome 2 [37]	CASP	14	7	5	2	Moderate	Include
Sørensen et al., 2022, Denmark [38]	JBI	8	8	0	(#)	High	Include
Bigi et al., 2023, Italy–outcome 1 [39]	CASP	10	9	0	1	High	Include
Bigi et al., 2023, Italy–outcome 2 [39]	CASP	14	14	0	0	High	Include

Legend: (#) The JBI appraisal checklist does not include the item “Number of Can’t Tell”; instead, it uses “Number of Unclear” and “Number of Not Applicable”, both of which are scored as zero; CASP—Critical Appraisal Skills Programme; JBI—Joanna Briggs Institute; USA—United States of America.

## Data Availability

The original contributions presented in this study are included in the article/Supplementary Materials. Further inquiries can be directed to the corresponding author.

## Supplementary Materials

The following supporting information can be downloaded at: https://www.mdpi.com/article/10.3390/healthcare13212789/s1, Table S1: Database search strategies.

## List of Abbreviations and Acronyms

The following abbreviations are used in this manuscript:

CASP—Critical Appraisal Skills Programme; ED—Emergency Department; EOL—End-of-Life; EPC—Early Palliative Care; e-PRO—electronic Patient-Reported Outcomes; HCC—Healthcare Cost; HCU—Healthcare Utilization; HM—Hematologic Malignancy; ICU—Intensive Care Unit(s); MM—Multiple Myeloma; NSCLC—Non-Small Cell Lung Cancer; PC—Palliative Care; POD—Place of Death; QOL—Quality of Life; RCT—Randomized Controlled Trial(s); SCB—Symptom Control/Burden.

## References

[1] Tietsche de Moraes Hungria V., Chiattone C., Pavlovsky M., Abenoza L.M., Agreda G.P., Armenta J., Arrais C., Avendaño Flores O., Barroso F., Basquiera A.L. (2019). Epidemiology of Hematologic Malignancies in Real-World Settings: Findings from the Hemato-Oncology Latin America Observational Registry Study. J. Glob. Oncol..

[2] El-Jawahri A., Nelson A.M., Gray T.F., Lee S.J., LeBlanc T.W. (2020). Palliative and End-of-Life Care for Patients With Hematologic Malignancies. J. Clin. Oncol..

[3] Ebert R.P.C., Magnus M.M., Toro P., Manoel F.G., Costa F.F., Olalla Saad S.T., de Melo Campos P. (2023). Hematologic Malignancies Patients Face High Symptom Burden and Are Lately Referred to Palliative Consultation: Analysis of a Single Center Experience. Am. J. Hosp. Palliat. Med..

[4] Howell D.A., Shellens R., Roman E., Garry A.C., Patmore R., Howard M.R. (2011). Haematological malignancy: Are patients appropriately referred for specialist palliative and hospice care? A systematic review and meta-analysis of published data. Palliat. Med..

[5] Koumakis L., Schera F., Parker H., Bonotis P., Chatzimina M., Argyropaidas P., Zacharioudakis G., Schäfer M., Kakalou C., Karamanidou C. (2021). Fostering Palliative Care Through Digital Intervention: A Platform for Adult Patients with Hematologic Malignancies. Front. Digit. Health.

[6] LeBlanc T.W., El-Jawahri A. (2015). When and why should patients with hematologic malignancies see a palliative care specialist?. Hematol. Am. Soc. Hematol. Educ. Program.

[7] Dasch B., Kalies H., Feddersen B., Ruderer C., Hiddemann W., Bausewein C. (2017). Care of cancer patients at the end of life in a German university hospital: A retrospective observational study from 2014. PLoS ONE.

[8] Hui D., Didwaniya N., Vidal M., Shin S.H., Chisholm G., Roquemore J., Bruera E. (2014). Quality of end-of-life care in patients with hematologic malignancies: A retrospective cohort study. Cancer.

[9] Hui D., Kim S.H., Roquemore J., Dev R. (2014). Impact of timing and setting of palliative care referral on quality of end-of-life care in cancer patients. Cancer.

[10] LeBlanc T.W., Roeland E.J., El-Jawahri A. (2017). Early Palliative Care for Patients with Hematologic Malignancies: Is It Really so Difficult to Achieve?. Curr. Hematol. Malig. Rep..

[11] Rodrigo-Troyano A., Alonso A., Barril S., Fariñas O., Güell E., Pascual A., Castillo D. (2022). Impact of Early Referral to Palliative Care in Patients with Interstitial Lung Disease. J. Palliat. Med..

[12] Bassi I., Pastorello S., Guerrieri A., Giancotti G., Cuomo A.M., Rizzelli C., Coppola M., Valenti D., Nava S. (2024). Early palliative care program in idiopathic pulmonary fibrosis patients favors at-home and hospice deaths, reduces unplanned medical visits, and prolongs survival: A pilot study. Eur. J. Intern. Med..

[13] Cartoni C., Breccia M., Giesinger J.M., Baldacci E., Carmosino I., Annechini G., Palumbo G., Armiento D., Niscola P., Tendas A. (2021). Early Palliative Home Care versus Hospital Care for Patients with Hematologic Malignancies: A Cost-Effectiveness Study. J. Palliat. Med..

[14] Srinivasan V.J., Akhtar S., Huppertz J.W., Sidhu M., Coates A., Knudsen N. (2023). Prospective Cohort Study on the Impact of Early Versus Late Inpatient Palliative Care on Length of Stay and Cost of Care. Am. J. Hosp. Palliat. Med..

[15] Barnes A., Woodman R.J., Kleinig P., Briffa M., To T., Wigg A.J. (2020). Hepatobiliary and Pancreatic: Early palliative care referral in patients with end-stage liver disease is associated with reduced resource utilization. J. Gastroenterol. Hepatol..

[16] Kenny P., Liu D., Fiebig D., Hall J., Millican J., Aranda S., van Gool K., Haywood P. (2024). Specialist Palliative Care and Health Care Costs at the End of Life. PharmacoEconomics Open.

[17] Seow H., Barbera L.C., McGrail K., Burge F., Guthrie D.M., Lawson B., Chan K.K.W., Peacock S.J., Sutradhar R. (2022). Effect of Early Palliative Care on End-of-Life Health Care Costs: A Population-Based, Propensity Score–Matched Cohort Study. JCO Oncol. Pract..

[18] Potenza L., Scaravaglio M., Fortuna D., Giusti D., Colaci E., Pioli V., Morselli M., Forghieri F., Bettelli F., Messerotti A. (2024). Early palliative/supportive care in acute myeloid leukaemia allows low aggression end-of-life interventions: Observational outpatient study. BMJ Support. Palliat. Care.

[19] Bevins J., Bhulani N., Goksu S.Y., Sanford N.N., Gao A., Ahn C., Paulk M.E., Terauchi S., Pruitt S.L., Tavakkoli A. (2021). Early Palliative Care Is Associated with Reduced Emergency Department Utilization in Pancreatic Cancer. Am. J. Clin. Oncol..

[20] Mós J.R., Reis-Pina P. (2025). Early Integration of Palliative Care in Nononcological Patients: A Systematic Review. J. Pain Symptom Manag..

[21] Moreno-Alonso D., Porta-Sales J., Monforte-Royo C., Trelis-Navarro J., Sureda-Balarí A., Fernández De Sevilla-Ribosa A. (2018). Palliative care in patients with haematological neoplasms: An integrative systematic review. Palliat. Med..

[22] LeBlanc T.W., O’Donnell J.D., Crowley-Matoka M., Rabow M.W., Smith C.B., White D.B., Tiver G.A., Arnold R.M., Schenker Y. (2015). Perceptions of Palliative Care Among Hematologic Malignancy Specialists: A Mixed-Methods Study. J. Oncol. Pract..

[23] Chan K.Y., Gill H., Chan T.S.Y., Li C.W., Tsang K.W., Au H.Y., Wong C.Y., Hui C.H. (2021). Early integrated palliative care for haematology cancer patients—The impact on symptom burden in Hong Kong. Ann. Palliat. Med..

[24] Higgins J., Thomas J., Chandler J., Cumpston M., Li T., Page M., Welch V.A., Flemyng E. (2024). Cochrane Handbook for Systematic Reviews of Interventions Version 6.5.

[25] Page M.J., McKenzie J.E., Bossuyt P.M., Boutron I., Hoffmann T.C., Mulrow C.D., Shamseer L., Tetzlaff J.M., Akl E.A., Brennan S.E. (2021). The PRISMA 2020 statement: An updated guideline for reporting systematic reviews. Br. Med. J..

[26] Elliott E., Watson T., Singh D., Wong C., Lo S.S. (2021). Outcomes of Specialty Palliative Care Interventions for Patients with Hematologic Malignancies: A Systematic Review. J. Pain Symptom Manag..

[27] Sterne J.A.C., Savović J., Page M.J., Elbers R.G., Blencowe N.S., Boutron I., Cates C.J., Cheng H.-Y., Corbett M.S., Eldridge S.M. (2019). RoB 2: A revised tool for assessing risk of bias in randomised trials. Br. Med. J..

[28] Sterne J., Higgins J. (2024). ROBINS-I V2: Risk of Bias in Non-Randomized Studies—Of Interventions, Version 2. https://www.riskofbias.info/welcome/robins-i-v2.

[29] CASP—Critical Appraisal Skills Programme (2020). CASP Checklist: CASP Cohort Study Checklist. https://casp-uk.net/casp-tools-checklists/cohort-study-checklist/.

[30] CASP—Critical Appraisal Skills Programme (2020). CASP Checklist: CASP Cross-Sectional Studies Checklist. https://casp-uk.net/casp-tools-checklists/cross-sectional-studies-checklist/.

[31] CASP—Critical Appraisal Skills Programme (2020). CASP Checklist: CASP Qualitative Studies Checklist. https://casp-uk.net/casp-tools-checklists/qualitative-studies-checklist/.

[32] Joanna Briggs Institute (2020). JBI Critical Appraisal Checklist for Case Reports. https://jbi.global/critical-appraisal-tools.

[33] Henckel C., Revette A., Huntington S.F., Tulsky J.A., Abel G.A., Odejide O.O. (2020). Perspectives Regarding Hospice Services and Transfusion Access: Focus Groups with Blood Cancer Patients and Bereaved Caregivers. J. Pain Symptom Manag..

[34] Jackson I., Etuk A., Jackson N. (2023). Prevalence and Predictors of Palliative Care Utilization among Hospitalized Patients with Diffuse Large B-Cell Lymphoma. J. Palliat. Care.

[35] Richter J., Sanchez L., Biran N., Wang C.K., Tanenbaum K., DeVincenzo V., Grunman B., Vesole D.H., Siegel D.S., Pecora A. (2021). Prevalence and Survival Impact of Self-Reported Symptom and Psychological Distress Among Patients with Multiple Myeloma. Clin. Lymphoma Myeloma Leuk..

[36] Samala R.V., Nurse D.P., Chen X., Wei W., Crook J.J., Fada S.D., Valent J. (2024). Effects of early palliative care integration on patients with newly diagnosed multiple myeloma. Support. Care Cancer.

[37] Weisse C.S., Melekis K., Cheng A., Konda A.K., Major A. (2024). Mixed-Methods Study of End-of-Life Experiences of Patients with Hematologic Malignancies in Social Hospice Residential Home Care Settings. JCO Oncol. Pract..

[38] Sørensen J., Sørensen T.V., Andersen K.H., Nørøxe A.D.S., Mylin A.K. (2022). Early, Patient-Centered, and Multidisciplinary Approach in Newly Diagnosed Multiple Myeloma: What Are We Talking About? A Case Description and Discussion. Palliat. Med. Rep..

[39] Bigi S., Borelli E., Potenza L., Gilioli F., Artioli F., Porzio G., Luppi M., Bandieri E. (2023). Early palliative care for solid and blood cancer patients and caregivers: Quantitative and qualitative results of a long-term experience as a case of value-based medicine. Front. Public Health.

[40] Vergnenègre A., Hominal S., Tchalla A.E., Bérard H., Monnet I., Fraboulet G., Baize N., Audigier-Valette C., Robinet G., Oliviero G. (2013). Assessment of palliative care for advanced non-small-cell lung cancer in France: A prospective observational multicenter study (GFPC 0804 study). Lung Cancer.

[41] Nottelmann L., Groenvold M., Vejlgaard T.B., Petersen M.A., Jensen L.H. (2021). Early, integrated palliative rehabilitation improves quality of life of patients with newly diagnosed advanced cancer: The Pal-Rehab randomized controlled trial. Palliat. Med..

[42] Temel J.S., Sloan J., Zemla T., Greer J.A., Jackson V.A., El-Jawahri A., Kamdar M., Kamal A., Blinderman C.D., Strand J. (2020). Multisite, Randomized Trial of Early Integrated Palliative and Oncology Care in Patients with Advanced Lung and Gastrointestinal Cancer: Alliance A221303. J. Palliat. Med..

[43] Sanders J.J., Temin S., Ghoshal A., Alesi E.R., Ali Z.V., Chauhan C., Cleary J.F., Epstein A.S., Firn J.I., Jones J.A. (2024). Palliative Care for Patients with Cancer: ASCO Guideline Update. J. Clin. Oncol..

[44] Pantilat S.Z., O’Riordan D.L., Dibble S.L., Landefeld C.S. (2010). Hospital-based palliative medicine consultation: A randomized controlled trial. Arch. Intern. Med..

[45] Gade G., Venohr I., Conner D., McGrady K., Beane J., Richardson R.H., Williams M.P., Liberson M., Blum M., Della Penna R. (2008). Impact of an inpatient palliative care team: A randomized control trial. J. Palliat. Med..

[46] Kircher C.E., Hanna T.P., Tranmer J., Goldie C.E., Ross-White A., Moulton E., Flegal J., Goldie C.L. (2025). Defining “early palliative care” for adults diagnosed with a life-limiting illness: A scoping review. BMC Palliat. Care.

[47] Sítima G., Galhardo-Branco C., Reis-Pina P. (2024). Equity of access to palliative care: A scoping review. Int. J. Equity Health.

[48] Reis-Pina P., Santos R.G. (2019). Early Referral to Palliative Care: The Rationing of Timely Health Care for Cancer Patients. Acta Medica Port..

[49] El-Jawahri A., Webb J.A., Hannon B., Zimmermann C. (2024). Integrating Palliative Care and Hematologic Malignancies: Bridging the Gaps for Our Patients and Their Caregivers. ASCO Educ. Book.

[50] Franjul Sánchez A., Fuentes Armesto A.M., Briones Chávez C., Ruiz M. (2020). Revisiting Early Palliative Care for Patients with Hematologic Malignancies and Bone Marrow Transplant: Why the Delay?. Cureus.

[51] Nickolich M., El-Jawahri A., LeBlanc T.W. (2016). Palliative and End-of-Life Care in Myelodysplastic Syndromes. Curr. Hematol. Malig. Rep..

[52] Taber J.M., Ellis E.M., Reblin M., Ellington L., Ferrer R.A. (2019). Knowledge of and beliefs about palliative care in a nationally-representative U.S. sample. PLoS ONE.

[53] Vanbutsele G., Deliens L., Cocquyt V., Cohen J., Pardon K., Chambaere K. (2019). Use and timing of referral to specialized palliative care services for people with cancer: A mortality follow-back study among treating physicians in Belgium. PLoS ONE.

[54] Wedding U. (2021). Palliative care of patients with haematological malignancies: Strategies to overcome difficulties via integrated care. Lancet Healthy Longev..

[55] Hui D., Park M., Liu D., Reddy A., Dalal S., Bruera E. (2015). Attitudes and Beliefs Toward Supportive and Palliative Care Referral Among Hematologic and Solid Tumor Oncology Specialists. Oncologist.

[56] Salins N., Ghoshal A., Hughes S., Preston N. (2020). How views of oncologists and haematologists impacts palliative care referral: A systematic review. BMC Palliat. Care.

[57] Temel J.S., Greer J.A., Muzikansky A., Gallagher E.R., Admane S., Jackson V.A., Dahlin C.M., Blinderman C.D., Jacobsen J., Pirl W.F. (2010). Early Palliative Care for Patients with Metastatic Non–Small-Cell Lung Cancer. N. Engl. J. Med..

[58] Chen M., Yu H., Yang L., Yang H., Cao H., Lei L., Ma L., Liu S., Tian L., Wang S. (2023). Combined early palliative care for non-small-cell lung cancer patients: A randomized controlled trial in Chongqing, China. Front. Oncol..

[59] Cui J., Fang P., Bai J., Tan L., Wan C., Yu L. (2023). Meta-Analysis of Effects of Early Palliative Care on Health-Related Outcomes Among Advanced Cancer Patients. Nurs. Res..

[60] Haroen H., Maulana S., Harun H., Mirwanti R., Sari C.W.M., Platini H., Arovah N.I., Padila P., Amirah S., Pardosi J.F. (2025). The benefits of early palliative care on psychological well-being, functional status, and health-related quality of life among cancer patients and their caregivers: A systematic review and meta-analysis. BMC Palliat. Care.

[61] Kim C.A., Lelond S., Daeninck P.J., Rabbani R., Lix L., McClement S., Chochinov H.M., Goldenberg B.A. (2023). The impact of early palliative care on the quality of life of patients with advanced pancreatic cancer: The IMPERATIVE case-crossover study. Support. Care Cancer.

[62] Shih H.-H., Chang H.-J., Huang T.-W. (2022). Effects of Early Palliative Care in Advanced Cancer Patients: A Meta-Analysis. Am. J. Hosp. Palliat. Med..

[63] Vanbutsele G., Van Belle S., Surmont V., De Laat M., Colman R., Eecloo K., Naert E., De Man M., Geboes K., Deliens L. (2020). The effect of early and systematic integration of palliative care in oncology on quality of life and health care use near the end of life: A randomised controlled trial. Eur. J. Cancer.

[64] Allende S., Turcott J.G., Verástegui E., Rodríguez-Mayoral O., Flores-Estrada D., Pérez Camargo D.A., Ramos-Ramírez M., Martínez-Hernández J.-N., Oñate-Ocaña L.F., Pina P.S. (2024). Early Incorporation to Palliative Care (EPC) in Patients with Advanced Non-Small Cell Lung Cancer: The PACO Randomized Clinical Trial. Oncologist.

[65] Epstein A.S., Goldberg G.R., Meier D.E. (2012). Palliative care and hematologic oncology: The promise of collaboration. Blood Rev..

[66] Allende-Pérez S., García-Salamanca M.F., Peña-Nieves A., Ramírez-Ibarguen A., Verástegui-Avilés E., Hernández-Lugo I., LeBlanc T.W. (2023). Palliative Care in Patients with Hematological Malignancies. We Have a Long Way to Go…. Am. J. Hosp. Palliat. Med..

[67] Giusti D., Colaci E., Pioli V., Banchelli F., Maccaferri M., Leonardi G., Marasca R., Morselli M., Forghieri F., Bettelli F. (2024). Early palliative care versus usual haematological care in multiple myeloma: Retrospective cohort study. BMJ Support. Palliat. Care.

[68] Haun M.W., Estel S., Rücker G., Friederich H.-C., Villalobos M., Thomas M., Hartmann M. (2017). with advanced cancer. Cochrane Database Syst. Rev..

[69] Bakitas M.A., Tosteson T.D., Li Z., Lyons K.D., Hull J.G., Li Z., Dionne-Odom J.N., Frost J., Dragnev K.H., Hegel M.T. (2015). Early Versus Delayed Initiation of Concurrent Palliative Oncology Care: Patient Outcomes in the ENABLE III Randomized Controlled Trial. J. Clin. Oncol..

