# Supplementary material for: Early Palliative Care in Advanced Hematologic Malignancies: A Systematic Review of Patient-Centered Outcomes
Source: Healthcare (Basel). 2025 Nov 3;13(21):2789. doi: 10.3390/healthcare13212789 (PMC12609357; doi:10.3390/healthcare13212789)
Supplement: Supplementary file 1 [file healthcare-13-02789-s001.zip › healthcare-3929152-supplementary.pdf]

Supplementary Table S1 - Database search strategies

Database	Search string	Limits/Filters
MEDLINE (via PubMed)	((("early"[All Fields] OR ("timely"[All Fields] OR "timing"[All Fields] OR "timings"[All Fields]) OR "refer*"[All Fields]) AND ("palliative care"[All Fields] OR "hospice care"[All Fields] OR "end of life care"[All Fields] OR "terminal care"[All Fields]) AND ("hematolog* malignanc*"[All Fields] OR "blood cancer*"[All Fields] OR "bone marrow cancer"[All Fields] OR ("leukaemia"[All Fields] OR "leukemia"[MeSH Terms] OR "leukemia"[All Fields] OR "leukaemias"[All Fields] OR "leukemias"[All Fields] OR "leukemia s"[All Fields]) OR ("lymphoma"[MeSH Terms] OR "lymphoma"[All Fields] OR "lymphomas"[All Fields] OR "lymphoma s"[All Fields]) OR ("multiple myeloma"[MeSH Terms] OR ("multiple"[All Fields] AND "myeloma"[All Fields]) OR "multiple myeloma"[All Fields] OR "myeloma"[All Fields] OR "myelomas"[All Fields] OR "myeloma s"[All Fields]))) NOT ("review"[Publication Type] OR "review literature as topic"[MeSH Terms] OR "review"[All Fields]))	MEDLINE; Humans; 2020/9/11 - 2024/7/19; English; All adult
Web of Science Core Collection	(early OR timely OR refer*) (Topic) AND ("palliative care" OR "hospice care" OR "end of life care" OR "terminal care") (Topic) AND ("hematolog* malignanc*" OR "blood cancer*" OR "bone marrow cancer" OR leukemia OR lymphoma OR myeloma) (Topic) NOT REVIEW (Topic) NOT child* or pediatr* (Topic)	2020/9/11 - 2024/7/19; English
Scopus	(TITLE-ABS-KEY (("palliative care" OR "hospice care" OR "end of life care" OR "terminal care")) AND TITLE-ABS-KEY ((early OR timely OR refer*)) AND TITLE-ABS-KEY (("hematolog* malignanc*" OR "blood cancer*" OR "bone marrow cancer" OR leukemia OR lymphoma OR myeloma)) AND NOT TITLE-ABS-KEY (review))	2020–2024; English: Human; Adult
Cochrane Library	("palliative care" OR "hospice care" OR "end of life care" OR "terminal care") in Title Abstract Keyword AND (early OR timely OR refer*) in Title Abstract Keyword AND ("hematolog* malignanc*" OR "blood cancer*" OR "bone marrow cancer" OR leukemia OR lymphoma OR myeloma) in Title Abstract Keyword NOT review in Title Abstract Keyword NOT "Child" in Title Abstract Keyword	Sep 2020 and Jul 2024; English
